# Effects and Mechanisms of Stevioside on Metabolic Dysfunction‐Associated Steatotic Liver Disease

**DOI:** 10.1002/fsn3.71945

**Published:** 2026-05-27

**Authors:** Kangjun Li, Changfa Zhang, Lili Kang, Yunping Mu, Zemin Li, Jingyi Zhang, Guowei Li

**Affiliations:** ^1^ Center for Clinical Epidemiology and Methodology (CCEM) The Affiliated Guangdong Second Provincial General Hospital of Jinan University Guangzhou China; ^2^ School of Biomedical and Pharmaceutical Sciences Guangdong University of Technology Guangzhou China; ^3^ School of Public Health Guangdong Pharmaceutical University Guangzhou China; ^4^ Father Sean O'Sullivan Research Centre St Joseph's Healthcare Hamilton Hamilton Ontario Canada

**Keywords:** lipid metabolism, liver fibrosis, MASLD, nonnutritive sweetener, oxidative stress, steatohepatitis, steviol glycosides, stevioside

## Abstract

Metabolic dysfunction‐associated steatotic liver disease (MASLD) affects more than 30% of adults globally and has emerged as a significant public health concern. Stevioside (SV), as a noncaloric sweetener derived from natural sources, has been reported to have beneficial effects on MASLD in several studies. Given that SV is widely used as a food sweetener, clarifying its effects on MASLD is of importance for controlling the increasing disease burden of MASLD. However, the hepatoprotective effects and molecular mechanisms of SV remain inconclusive, highlighting the need for a comprehensive review to summarize the best available evidence. We therefore conducted a systematic review of preclinical and clinical studies to critically assess the effects of SV on MASLD and to evaluate the underlying mechanisms in detail. We performed a comprehensive search of PubMed and Web of Science (up to February 20, 2025) and included a total of two clinical trials, 36 animal studies, and two cellular studies for review. Results from preclinical studies demonstrated that SV could prevent or treat MASLD by improving hepatic lipid metabolism, enhancing anti‐inflammatory and antioxidant capacity, and attenuating hepatic fibrosis. Yet the evidence from clinical trials regarding the beneficial effects of SV on MASLD was sparse and inconsistent, requiring further exploration. In conclusion, SV represents a promising dietary strategy for the potential prevention and treatment of MASLD, provided its hepatoprotective effects are validated in clinical trials and population studies.

## Graphical abstract

SV has the potential to prevent or treat MASLD by improving hepatic lipid metabolism, enhancing anti‐inflammatory and antioxidant capacity, and attenuating hepatic fibrosis.

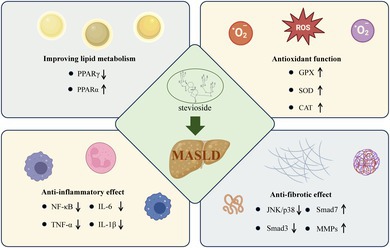

**Abbreviations**
MASLD: metabolic dysfunction‐associated steatotic liver disease
NF‐κB: nuclear factor kappa‐light‐chain‐enhancer of activated B cells
NRF2: nuclear factor erythroid 2‐related factor 2
PPARs: peroxisome proliferator‐activated receptors
SV: stevioside
TGF‐β: transforming growth factor‐beta

## 1 Introduction

Metabolic dysfunction‐associated steatotic liver disease (MASLD) is a chronic metabolic disease characterized by excessive fat accumulation in the liver (Zhang and Brandman 2025). MASLD encompasses a spectrum of liver abnormalities, including steatosis, metabolic dysfunction‐associated steatohepatitis, fibrosis, and cirrhosis (Rinella et al. 2023; Sawada et al. 2023). It is estimated that over 30% of adults are affected by MASLD globally, a prevalence that has risen substantially from 17.6% in 1990 to 23.4% in 2019 (Miao et al. 2024). The diagnostic approaches for MASLD mainly include liver biopsy and noninvasive assessments; however, liver biopsy is invasive and costly, with poor acceptability (Kang et al. 2021). The noninvasive assessments primarily rely on either a “biological” approach that is based on biomarkers in serum samples or a “physical” approach that directly measures liver stiffness (Castera et al. 2019). Nevertheless, both of them have notable shortcomings including suboptimal diagnostic accuracy, susceptibility to interindividual variability, and inability to provide dynamic monitoring of disease progression (Abdelhameed et al. 2024). The rising prevalence and marked diagnostic challenges of MASLD not only pose a substantial economic burden but also associate with significant increases in end‐stage liver disease and liver cancer (Goldberg et al. 2017; Terrault et al. 2023).

The central pathogenic mechanism of MASLD involves the accumulation of excess free fatty acids in the liver (Bansal and Bansal 2024), which drives de novo lipogenesis and free fatty acid production largely due to excessive carbohydrate intake (Bo et al. 2024). Excess fatty acids generate lipotoxic metabolites, trigger endoplasmic reticulum stress, mitochondrial dysfunction and oxidative stress, and ultimately promote inflammatory cytokine overexpression (Guy et al. 2012; Han and Kaufman 2016). This cascade further triggers the activation of hepatic stellate cells and accumulation of extracellular matrix, eventually accelerating the progression to liver fibrosis (Bruschi et al. 2017).

While lifestyle modification remains the cornerstone of MASLD management, long‐term adherence is often challenging, and no pharmacological therapies have yet received widespread regulatory approval (Drygalski 2025). Recent research has identified some promising therapeutic agents, including PPAR agonists, FXR agonists, and GLP‐1 receptor agonists; however, the current prevention and treatment of MASLD in clinical practice remain unsatisfactory (Sumida and Yoneda 2018). Long‐term consumption of high‐sugar foods and beverages is well recognized as a major dietary cause of MASLD (Tsompanaki et al. 2023). Consequently, nonnutritive sweeteners have been increasingly used as sugar substitutes that provide a sweet taste with few or no calories (Fernstrom 2015). However, safety concerns about nonnutritive artificial sweeteners have been continuously raised, including elevated risks of impaired glucose tolerance, obesity, diabetes, cardiovascular disease, and cancers (Malik 2019).

*Stevia rebaudiana* Bertoni, originating from South America, has been used as a natural sweetener for years without reported safety concerns. The sweetness components of *Stevia rebaudiana* Bertoni are steviol glycosides that include stevioside (SV), rebaudioside A, rebaudioside D, rebaudioside M, and other minor components. Among them, SV is the most abundant compound of *Stevia rebaudiana* Bertoni and is approximately 300 times sweeter than sucrose (Chatsudthipong and Muanprasat 2009; Hanson and De Oliveira 1993). Recently, SV has been reported to have beneficial effects on MASLD, mainly due to its anti‐inflammatory, antioxidant, and anti‐fibrotic properties (Huang et al. 2024). However, the effects of SV on MASLD in humans remain inconclusive, as evidence from clinical and population studies is sparse and limited (Orellana‐Paucar 2023). Similarly, the effects and mechanisms of SV on MASLD from preclinical studies require further elucidation, especially given some in vivo studies demonstrating no hepatoprotective effects of SV (Ghanta et al. 2007). This necessitates a comprehensive and critical review to summarize the best available evidence and assess the effects and mechanisms of SV on MASLD.

Therefore, we conducted a systematic review of clinical and preclinical studies to critically evaluate the effects of SV on MASLD and elucidate the underlying mechanisms. Findings may help clarify the hepatoprotective effects and molecular mechanisms of SV on MASLD, thereby providing new insights into potential prevention and treatment of MASLD from the perspective of natural sweeteners.

## 2 Methods

We systematically searched PubMed and Web of Science (from inception to February 20, 2025) using the strategies detailed in Table S1. We searched PubMed by combining MeSH terms and title/abstract keywords for stevia compounds (e.g., “stevia”[Mesh], stevia*[Title/Abstract], “stevioside”[Supplementary Concept]) and liver‐related outcomes (e.g., “Non‐alcoholic fatty liver disease”[Mesh], “Lipid metabolism”[Mesh], “Oxidative stress”[Mesh], “liver”[Title/Abstract]). For Web of Science, topic searches were performed using equivalent terms. The complete search strings, including Boolean operators and record counts for each step, are provided in Table S1. This systematic review was conducted in accordance with the PRISMA 2020 guidelines, and the completed PRISMA 2020 checklist is provided as Data S1. We also manually searched the reference lists of related reviews to identify potentially eligible studies. Inclusion criteria were (i) clinical studies (including trials) and preclinical evidence (including in vivo and in vitro studies) assessing the effects of SV on MASLD, and (ii) being published in English. Some studies may not treat MASLD as their primary outcome, but provided data on lipid metabolism and hepatic functions related to metabolic conditions. To collect relevant evidence comprehensively, these studies were also included for review. Similarly, we also considered studies that used stevia extracts or other steviol glycosides (e.g., rebaudioside A, D, or M), as they all share the common steviol skeleton (Lemus‐Mondaca et al. 2012). It should be noted that these were included as a supplementary exploration and not as part of the primary analysis focused solely on SV. We only included studies in English because we aimed to reach readers worldwide and enhance the reproducibility of our work. Reviews, abstracts, and conference papers were excluded.

**FIGURE 1:**
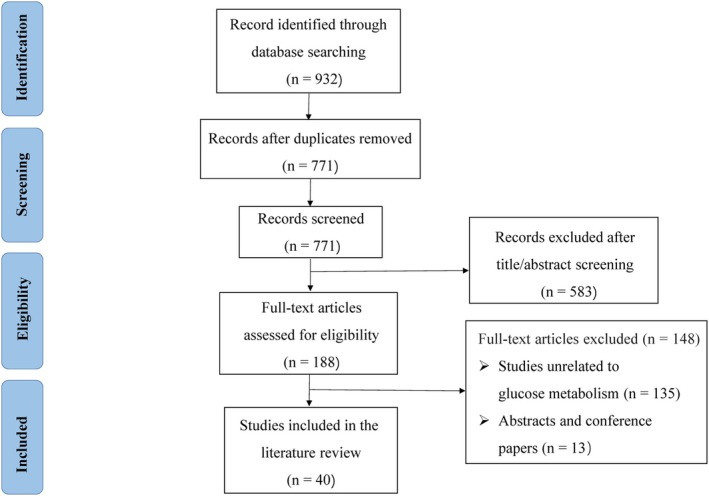
Flow diagram of study selection process.

Two study authors (K.L. and L.K.) independently screened and determined whether studies were eligible for inclusion. Duplicate records were first removed by the reference management software (EndNote) before title and abstract screening. Full texts of potentially relevant articles were then independently assessed against the predefined inclusion criteria. Any disagreements during the screening or full‐text assessment were resolved through discussion and, if necessary, by consultation with a third reviewer (G.L.). For each included study, these two reviewers independently extracted the following data using a standardized data extraction form: first author, year of publication, study design (clinical trial, animal study, or in vitro study), characteristics of the model (species, strain, sex, sample size, induction method for MASLD), intervention details (type and purity of steviol glycosides, dose, route, duration), comparator(s), and main outcomes related to MASLD (including hepatic lipid parameters, markers of oxidative stress, inflammatory markers, and fibrosis indicators). Any discrepancies in data extraction were resolved by discussion or by another reviewer (G.L.). A total of two clinical trials and 38 preclinical studies (36 animal and 2 cell studies) were included for this review (Figure 1 presents the selection process and Table S2 shows the detailed included studies). We assessed the risk of bias of the two included clinical trials using the Cochrane risk of bias (RoB) 2.0 tool.

**FIGURE 2:**
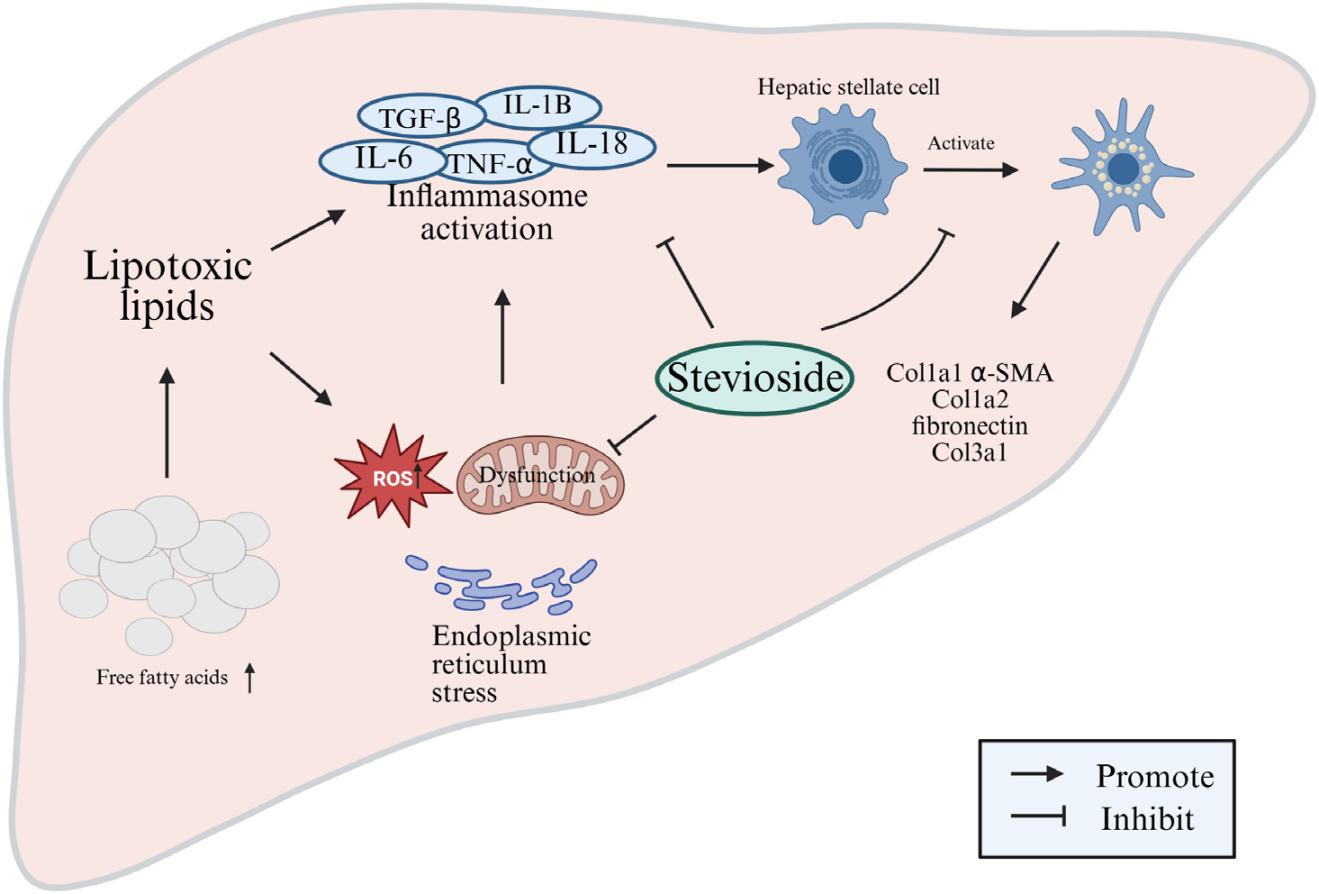
The potential effects of SV on MASLD. The core pathogenesis of MASLD lies in the accumulation of free fatty acids. Excessive free fatty acids lead to the generation of lipotoxic species, which induce endoplasmic reticulum stress, oxidative stress, and the production of inflammatory cytokines. This cascade results in the activation of hepatic stellate cells, overexpress collagen and fibronectin, promoting the accumulation of extracellular matrix components, and eventually causing liver fibrosis. On the other hand, SV helps improve MASLD by enhancing the antioxidant defense system, inhibiting pro‐inflammatory signaling pathways, and attenuating liver fibrosis.

In this review, we first evaluated the effects of SV on MASLD from clinical studies. Subsequently, we summarized the effects and mechanisms of SV on MASLD from preclinical studies, where the mechanisms included the improvement of lipid metabolism, augmentation of antioxidant capacity, mitigation of hepatic inflammation, reduction of hepatic fibrosis, and others (Figure 2). Of note, we identified three intervention types throughout this review, including pure SV (as our primary focus), stevia leaf extracts (mixtures of steviol glycosides and other plant constituents), and other individual steviol glycosides (e.g., rebaudioside A or M). As intervention purity can influence hepatoprotective effects, we explicitly displayed the types to avoid conflating with findings from pure SV when discussing effects of other extracts or glycosides on MASLD.

## 3 Effects of SV on MASLD From Clinical Studies

We only included two clinical trials evaluating the effects of SV on liver function in patients with metabolic conditions for review, both demonstrating the safety and potential hepatoprotective properties of SV (Table 1). In the study by Zafrilla et al. (2021), 136 overweight participants without chronic diseases or smoking history were enrolled. Participants were randomly allocated into three groups: a sucrose group, a stevia group (consuming citrus‐maqui juice containing 4 mg/100 mL of stevia), and a sucralose group, with their mean ages ranging from 42 to 44 years. All three groups consumed 330 mL of the juice daily, and the beverages were matched for sweetness. Following a 60‐day intervention period, participants' hepatic biomarkers (alkaline phosphatase, alanine aminotransferase, aspartate aminotransferase, γ‐glutamyl transferase) and total bilirubin levels were assessed and compared with pre‐intervention measurements. Biochemical analyses revealed that the stevia group experienced a 9% reduction in alkaline phosphatase levels, and no statistically significant changes in other hepatic enzymes or bilirubin concentrations. This indicated no evidence of hepatotoxicity for stevia. Moreover, while stevia had no significant impact on lipid metabolism parameters, it significantly enhanced oxygen radical absorbance capacity in participants with low baseline antioxidant capacity. In addition, stevia increased the production of anti‐inflammatory cytokine IL‐10, with 22% of participants demonstrating a fourfold increase in circulating IL‐10 levels compared to baseline. These results suggested the potential of stevia to mitigate oxidative stress and inflammation in overweight adults.

**TABLE 1:** Effects of SV on MASLD from clinical trials.

Trial ID	Population	Intervention	Duration of follow‐up	Outcome	Main finding	MASLD‐specific endpoints (imaging/biopsy)
Zafrilla et al. (2021)	136 overweight participants (BMI 24.9–29.9 kg/m^2^), 41% female; aged 35–55 years. No data on liver condition at baseline.	Stevia (4 mg/100 mL; *n* = 45); sucralose (4 mg/100 mL; *n* = 46); sucrose (7.5 g/100 mL; *n* = 45). Participants drank 330 mL juices containing SV/sucralose/sucrose everyday for 60 days. Stevia is delivered in a juice matrix (potential confounding).	60 days	Lipid profile (total cholesterol, LDL‐cholesterol, HDL‐cholesterol, and triglycerides); hepatic safety parameters (alkaline phosphatase, alanine aminotransferase, aspartate aminotransferase, γ‐glutamyl transferase, and total bilirubin); antioxidant status (oxygen radical absorbance capacity, homocysteine, and oxidized LDL); inflammatory biomarkers (IL‐6, TNF‐α, IL‐10).	Stevia had no effect on lipid profiles. Stevia had no effect on hepatotoxicity. Stevia increased IL‐10 and reduced oxidative stress. Stevia increased oxygen radical absorbance capacity in subjects with baseline oxygen radical absorbance capacity below population mean.	No
Almiron‐Roig et al. (2023)	60 overweight/obese participants (BMI 25–35 kg/m^2^), 47% female; mean age: 32.1 (SD 11.0). No data on liver condition at baseline.	Stevia rebaudioside A (24 mg/100 mL) + thaumatin (0.12 mg/100 mL); stevia rebaudioside M (20 mg/100 mL) + mogroside V (40 mg/100 mL); sucrose (10 mg/100 mL) + acesulfame‐potassium (10 mg/100 mL); sucrose (8 g/100 mL). Participant drank one beverage (330 mL) containing one group of sweeteners (with rebaudioside A and rebaudioside M mixed with other sweeteners) each time, and after 6–10 days drank another beverage containing another group of sweeteners. All participants finished all the four beverages at the end of the trial.	4–6 weeks	Fatty liver index; hepatic safety parameters (alkaline phosphatase, alanine aminotransferase); glycemic response (glucose/insulin iAUC); appetite (visual analogue scale); 24‐h energy intake; lipid profile.	SV had no significant alterations in fatty liver index scores. SV had no effect on liver function. Stevia rebaudioside A and M groups reduced glucose/insulin iAUC vs. sucrose. No differences in appetite, lipid profiles, and energy intake among the four groups.	No

Abbreviations: BMI, body mass index; IAUC, area under the curve incremental.

Another multinational trial involving 60 overweight or obese participants from the UK, Denmark, and Spain investigated acute responses to beverages containing stevia rebaudioside A (24 mg/100 mL) or rebaudioside M (20 mg/100 mL) over a 4‐ to 6‐week period (Almiron‐Roig et al. 2023). It is noteworthy that the doses of steviol glycosides employed in this trial exceeded the acceptable daily intake of 0–4 mg/kg body weight/day (expressed as steviol equivalents) established by the Joint FAO/WHO Expert Committee on Food Additives (Younes et al. 2022). Despite these elevated levels, no significant differences in fatty liver index were found between the groups, where the fatty liver index was calculated based on body mass index (BMI), waist circumference, serum triglycerides, and γ‐glutamyl transferase levels (Yip et al. 2023). This null finding may be partly attributable to the small sample size and the relatively short‐term (4–6 week) investigation. Importantly, the intervention beverages contained an additional sweetener (stevia rebaudioside A plus Thaumatin; stevia rebaudioside M plus Mogroside V), while control groups received sucrose blends. Consequently, this study design, which used high doses and sweetener mixtures, does not allow for the assessment of the isolated effects of Reb A or Reb M.

The divergent results are largely attributable to these design disparities. The study by Zafrilla et al. suggested potential benefits of long‐term, low‐dose stevia consumption within a functional beverage context for improving antioxidant and anti‐inflammatory markers in overweight individuals. The null finding on liver indices in the acute, high‐dose blend study by Almiron‐Roig et al. may reflect its shorter timeframe, use of sweetener mixtures, and the insensitivity of the chosen surrogate marker. Of note, there were unclear risks of selective reporting and randomization, leading to the overall unclear ROB for the included trials (Figure S1). Thus, the effects of SV on MASLD in human beings from clinical studies remain largely inconclusive and sparse, requiring further evidence to clarify the hepatoprotective effects of SV from clinical trials and population studies.

## 4 Effects and Mechanisms of SV on MASLD From Preclinical Studies

### 4.1 SV Exerted Hepatoprotective Effects via Improving Lipid Metabolism

High‐calorie diets and sedentary lifestyles synergistically exacerbate hepatic lipid accumulation, and excessive lipid accumulation is a crucial hallmark of MASLD (Leung et al. 2017). When the lipid overload exceeds the liver's adaptive capacity, toxic lipid intermediates are generated to induce lipotoxic injury, mitochondrial dysfunction, and fibrosis, thereby accelerating the MASLD progression (Loomba et al. 2021; Luukkonen et al. 2016; Perry et al. 2014).

Peroxisome proliferator‐activated receptors (PPARs) are well‐known to modulate multiple biological processes, including inflammation, lipid metabolism, and systemic energy homeostasis (Gross et al. 2017). Park et al. (2022) demonstrated that SV acts as a PPARα agonist. In male C57BL/6J db/db mice, oral SV administration (40 mg/kg BW/day) suppressed adipogenesis and promoted fatty acid oxidation by inhibiting PPARγ expression and activating PPARα. Concurrently, SV enhanced hepatic lipophagy and reduced lipid accumulation in the liver. These findings were further validated in HepG2 cells, where treatment with SV (100 μM) significantly upregulated lipophagy‐related genes (TFEB/CPT‐1) and enhanced hepatic lipid catabolism to alleviate steatosis, an effect dependent on PPARα activation. Another study corroborated this PPARα‐mediated regulation in Sprague–Dawley rats fed a high‐fat diet (HFD) and daily SV (150 mg/kg BW) (Jia et al. 2019). Moreover, results from different concentrations of SV (100–400 μM) demonstrated dose‐dependent suppression of lipid accumulation in oleic acid‐treated BRL cells. Of note, following the suppression of PPARα, SV could no longer enhance lipid metabolism, confirming its PPARα‐dependent mechanism.

Moreover, Holvoet et al. (2015) used histopathological and metabolomic analyses to identify PPARγ as a critical mediator for the anti‐steatotic effects of SV in ob/ob and LDLR^−/−^ mice administered SV (10 mg/kg BW/day). Similarly, the study by Kurek et al. (2020, 2023) reported that ultrahigh‐dose SV (2500 mg/kg BW) improved lipid metabolism in HFD‐fed male Wistar rats by downregulating PPARγ. However, SV may ameliorate hepatic lipid metabolism through upstream signaling‐mediated indirect regulation of PPARγ, rather than functioning as direct agonists (Ferreira et al. 2006).

There were two studies using stevia leaf extracts as a gross composite (including SV, rebaudioside A, rebaudioside M, rebaudioside D, and other minor components) for intervention (Aghajanyan et al. 2017; Park and Cha 2010). Notably, these two studies showed that stevia leaf extracts had lipid‐modulating effects comparable to those of SV. For instance, similar to SV, oral administration of stevia leaf extracts (100 mg/kg BW) ameliorated high glucose‐induced lipid metabolism disorders and dyslipidemia in plasma (Aghajanyan et al. 2017), where plasma lipid levels are well‐known to associate with lipid metabolism, lipid synthesis, and lipid transport from the liver (Li et al. 2021). Another in vivo research using stevia leaf extracts reported that these extracts could also enhance fatty acid oxidation via PPARα activation and suppress lipogenesis in diet‐induced obese mice (Park and Cha 2010). They also observed that stevia leaf extracts improved carnitine metabolism and demonstrated enhanced fatty acid oxidation in both mitochondria and peroxisomes.

Taken together, the majority of studies indicated that SV could reduce hepatic lipid accumulation by modulating PPARs pathways (Table 2). Yet, this effect of SV on MASLD showed substantial variability across models and doses.

**TABLE 2:** Effects and mechanisms of SV on improving lipid metabolism from preclinical studies.

Study ID	Experimental design details	Model	Main results	Potential mechanisms
Park et al. (2022)	Stevia extracts (200/500 mg/kg BW/day), SV (40 mg/kg BW/day) in mice	db/db mice (hepatic steatosis)	Reduced body and liver weight; decreased serum triglycerides and total cholesterol; reduced hepatic lipogenic proteins; increased PPARα expression, elevated LC3B levels.	Activation of PPARα‐mediated lipophagy pathways.
Jia et al. (2019)	SV (75–150 mg/kg BW/day) in rats	HFD rats (hepatic steatosis)	Reduced hepatic total cholesterol, triglycerides, and free fatty acid; improved steatosis scores; upregulated PPARα/CPT‐1A; downregulated SREBP‐1c/FASN/DGAT.	SV modulates lipid metabolism‐associated gene expression through PPARα.
Holvoet et al. (2015)	SV (10 mg/kg BW/day), rebaudioside A (12 mg/kg BW/day), steviol (5 mg/kg BW/day) in mice	ob/ob‐LDLR^−/−^ mice (hepatic steatosis)	Attenuated hepatic steatosis; enhanced fat oxidation and bile acid metabolism.	SV modulates lipid metabolism‐associated gene expression through PPARs.
Kurek et al. (2023)	SV (500/2500 mg/kg BW/day), rebaudioside A (500/2500 mg/kg BW/day) in rats	HFD‐streptozotocin rats (hepatic steatosis)	Regulation of the expression of hepatic lipid metabolism genes such as FASN, CEBPA, and PPARγ.	Inhibition of the PPARγ signaling pathway.
Kurek et al. (2020)	SV (500/2500 mg/kg BW/day), rebaudioside A (500/2500 mg/kg BW/day) in rats	HFD‐streptozotocin rats	Reduced serum triglycerides, total cholesterol, LDL‐C, and alanine aminotransferase.	Modulation of lipid metabolism through activation of PPARα pathways.
Ferreira et al. (2006)	Stevia extracts (20 mg/kg BW/day) vs. SV (5.5 mg/kg BW/day) in rats	Male wistar rats	Stevia leaves and SV had no effect on PPARγ pathway.	Not reported
Aghajanyan et al. (2017)	Stevia extracts (100 mg/kg BW/day) in rabbits	Stress‐induced hyperglycemic rabbits	Reduced serum triglycerides, total cholesterol, and LDL‐C; increased HDL‐C; restored hepatic glycogen content.	Promote lipid metabolism to regulate blood lipids.
Park and Cha (2010)	Stevia extracts (1 mL/kg BW/day) in mice	HFD mice	Decreased body weight gain and hepatic triglycerides content; increased CPT‐1A and PPARα expression.	PPARα activation promoting fatty acid β‐oxidation.

Abbreviations: CEBPA, CCAAT/enhancer‐binding protein alpha; CPT‐1A, carnitine palmitoyltransferase 1A; DGAT, diacylglycerol O‐acyltransferase; FASN, fatty acid synthase; HFD, high‐fat diet; LC3B, microtubule‐associated protein 1A/1B‐light chain 3B; PPARα, peroxisome proliferator‐activated receptor alpha; SREBP‐1c, sterol regulatory element‐binding protein 1c.

### 4.2 SV Enhanced Antioxidant Function of the Liver

Excess free fatty acids can overwhelm hepatic metabolic capacity, leading to excessive mitochondrial β‐oxidation and overproduction of reactive oxygen species, which can disrupt mitochondrial membrane integrity and impair electron transport chain function (Patterson et al. 2016; Sunny et al. 2011). Lipid overload further exacerbates endoplasmic reticulum stress to activate the unfolded protein response and amplify oxidative signaling pathways (Ratziu et al. 2005). Chronic oxidative stress depletes the endogenous antioxidant defense system (such as glutathione peroxidase, superoxide dismutase, and catalase), establishing a self‐reinforcing cycle that further exacerbates hepatic injury in MASLD (Greatorex et al. 2023).

The transcription factor nuclear factor erythroid 2‐related factor 2 (NRF2) is a central regulator of redox, metabolic, and protein homeostasis that intersects with many other signaling cascades (Dodson et al. 2019). The antioxidant effects of SV are closely associated with NRF2 signaling. In a thioacetamide‐induced rat model of oxidative stress and inflammation in metabolically compromised livers, SV administration (20 mg/kg BW) enhanced endogenous antioxidant defense mechanisms through NRF2 upregulation, including significant elevation of hepatic glutathione and glutathione peroxidase levels, concurrent suppression of lipid peroxidation, and reduced malondialdehyde concentrations (Casas‐Grajales, Ramos‐Tovar, et al. 2019). This result aligned with findings from Rotimi et al. (2018), where their study demonstrated a dose‐dependent antioxidant effect of SV, with maximal antioxidant effects observed at the highest dose of 50 mg/kg BW.

Similar effects were also found for stevia leaf extracts as a gross composite containing SV. Specifically, stevia leaf extracts (100 mg/kg BW) effectively mitigated oxidative stress in cirrhotic rats through NRF2 upregulation, normalizing reduced glutathione/oxidized glutathione ratios while suppressing malondialdehyde, 4‐hydroxynonenal, and lipid peroxidation products (Ramos‐Tovar, Flores‐Beltrán, et al. 2019; Ramos‐Tovar, Flores‐Beltrán, et al. 2018). El‐Hadary and Sitohy (2021) reported that stevia leaf extracts (300 mg/kg BW) were more potent in enhancing hepatic antioxidant capacity than glibenclamide (10 mg/kg BW). Moreover, dietary supplementation with stevia leaf extracts (20 mg/kg BW) significantly improved hepatic antioxidant status, including hepatic coenzyme Q10, lutein, and total carotenoids in male broilers (Pirgozliev et al. 2021; Tang et al. 2024). Likewise, a linear dose–response relationship was observed in weaned piglets, where stevia leaf extracts (100–300 mg/kg BW) enhanced hepatic total antioxidant capacity alongside superoxide dismutase and glutathione peroxidase activities (Liu et al. 2022; Xiong et al. 2025). This enhancement of hepatic antioxidant capacity demonstrated across multiple animal models may indicate the preventive potential of stevia leaf extracts against MASLD. Notably, while Vaško et al. (2014) reported elevated hepatic antioxidant capacity with stevia leaf extracts, they failed to observe a reduction in malondialdehyde or elevation in superoxide dismutase activity, as found in other investigations (Ranjbar et al. 2020; Saadi et al. 2024). A similar discrepancy was observed in glutathione/oxidized glutathione ratios. While one study did not normalize the abnormally low hepatic glutathione/oxidized glutathione ratio in diabetic rats (Shivanna et al. 2013), Ramos‐Tovar, Hernández‐Aquino, et al. (2018) reported a significant elevation. These inconsistencies may, at least in part, stem from variations in extraction protocols that could substantially influence bioactive components and retention of active constituents of stevia.

The direct free radical scavenging capacity of SV remains contentious. Both SV administered in vivo (250 mg/kg BW) and stevia leaf extracts tested in vitro (100 μg/mL) demonstrated notable radical‐neutralizing activity, particularly against nitric oxide, hydrogen peroxide, and superoxide anions (Latha et al. 2017; Vaško et al. 2014). In contrast, Ghanta et al. (2007) found that stevia leaf extracts could not scavenge hydroxyl radicals under equivalent experimental conditions. Notably, results from ex vivo experiments in isolated rat mitochondria displayed that SV administration (5 mM) could inhibit oxidative phosphorylation and respiratory chain enzyme activities (Kelmer Bracht et al. 1985). Nevertheless, these findings are compromised due to the inherent inability of ex vivo models to replicate the complex in vivo metabolic interactions, and the supraphysiological SV concentrations (5 mM) exceeding practical exposure levels.

**FIGURE 3:**
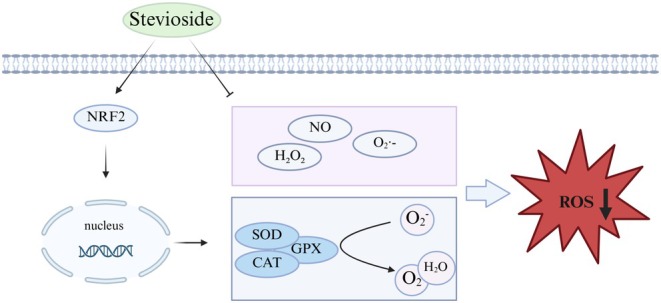
The potential antioxidant effect of SV on the liver. SV alleviates hepatic oxidative stress through dual mechanisms involving NRF2 signaling activation and direct free radical scavenging.

Therefore, SV was found to potentially alleviate hepatic oxidative stress through dual mechanisms involving NRF2 signaling activation and direct free radical scavenging (Figure 3). However, more evidence is needed for the standardized dosing protocols of SV and further clarification of the effects across various experimental models.

### 4.3 SV Improved the Anti‐Inflammatory Activity of Liver

In MASLD, excessive lipid accumulation produces cytotoxic metabolites, triggering a self‐perpetuating cycle of macromolecular damage, cellular stress, and inflammatory cascades (Kakisaka et al. 2012; Loomba et al. 2021). This process amplifies pro‐inflammatory cytokine production (including TNF‐α, IL‐6, and IL‐1β), promotes Kupffer cell polarization into pro‐inflammatory phenotypes, and induces neutrophil infiltration (Govaere et al. 2022; Kazankov et al. 2019; Zhang et al. 2019). Subsequently, the shift in the inflammatory response from type 1 to type 2 accelerates liver fibrosis. Therefore, enhancing the anti‐inflammatory activity of the liver could effectively inhibit further deterioration of MASLD.

The canonical nuclear factor kappa‐light‐chain‐enhancer of activated B cells (NF‐κB) pathway is primarily activated in response to inflammatory stimuli and serves as a pivotal regulator of pro‐inflammatory cytokines in MASLD (Luedde and Schwabe 2011). In a rat model of hepatitis, intraperitoneal administration of SV (20 mg/kg BW) significantly reduced both protein and mRNA expression levels of NF‐κB, inhibited its nuclear translocation, and diminished the release of downstream pro‐inflammatory mediators (IL‐1β, TNF‐α, IL‐6, IL‐17a), thereby effectively blocking inflammatory cascades (Casas‐Grajales, Ramos‐Tovar, et al. 2019). These anti‐inflammatory properties were further demonstrated in a coculture system of human hepatic stellate cells and VL‐17A cells, where SV treatment (20 mM) showed results consistent with in vivo observations. To elucidate the mechanistic underpinnings of SV's potential therapeutic effects, Casas‐Grajales et al. conducted computational molecular docking simulations and found substantial binding affinities between SV and the extracellular domains of TLR4/TNFR1. Subsequent structural analysis suggested that SV may mimic endogenous antagonists (e.g., Eritoran for TLR4 and IV703 for TNFR1) through analogous binding interactions, thereby blocking downstream inflammatory signaling. Of note, TLR4 and TNFR1, both serving as principal receptors for free fatty acids and TNF‐α respectively, can form a critical amplification loop that drives inflammatory progression through hepatic TNF‐α–TNFR1 signaling.

Evidence from two studies demonstrated that stevia leaf extracts (10–500 mg/kg BW) exhibited comparable anti‐inflammatory effects to SV (20 mg/kg BW) on rodent models (Latha et al. 2017; Ramos‐Tovar, Flores‐Beltrán, et al. 2019). Both stevia leaf extracts and SV similarly suppressed NF‐κB activation and reduced pro‐inflammatory cytokine expression in hepatocytes while restoring the compensatory elevation of anti‐inflammatory IL‐10 to physiological levels. Moreover, in diabetic MASLD rat models, stevia leaf extracts were found to promote the expression of IL‐10 rather than suppress it (Saadi et al. 2024). Mechanistically, this phenomenon may arise from IL‐10 depletion by chronic diabetes‐associated inflammatory burden combined with compromised functionality of hepatic T‐lymphocytes and other IL‐10‐producing immune cell populations. The administration of stevia extracts may reactivate anti‐inflammatory signaling cascades, thereby reconstituting IL‐10 homeostasis through multiple regulatory nodes.

**FIGURE 4:**
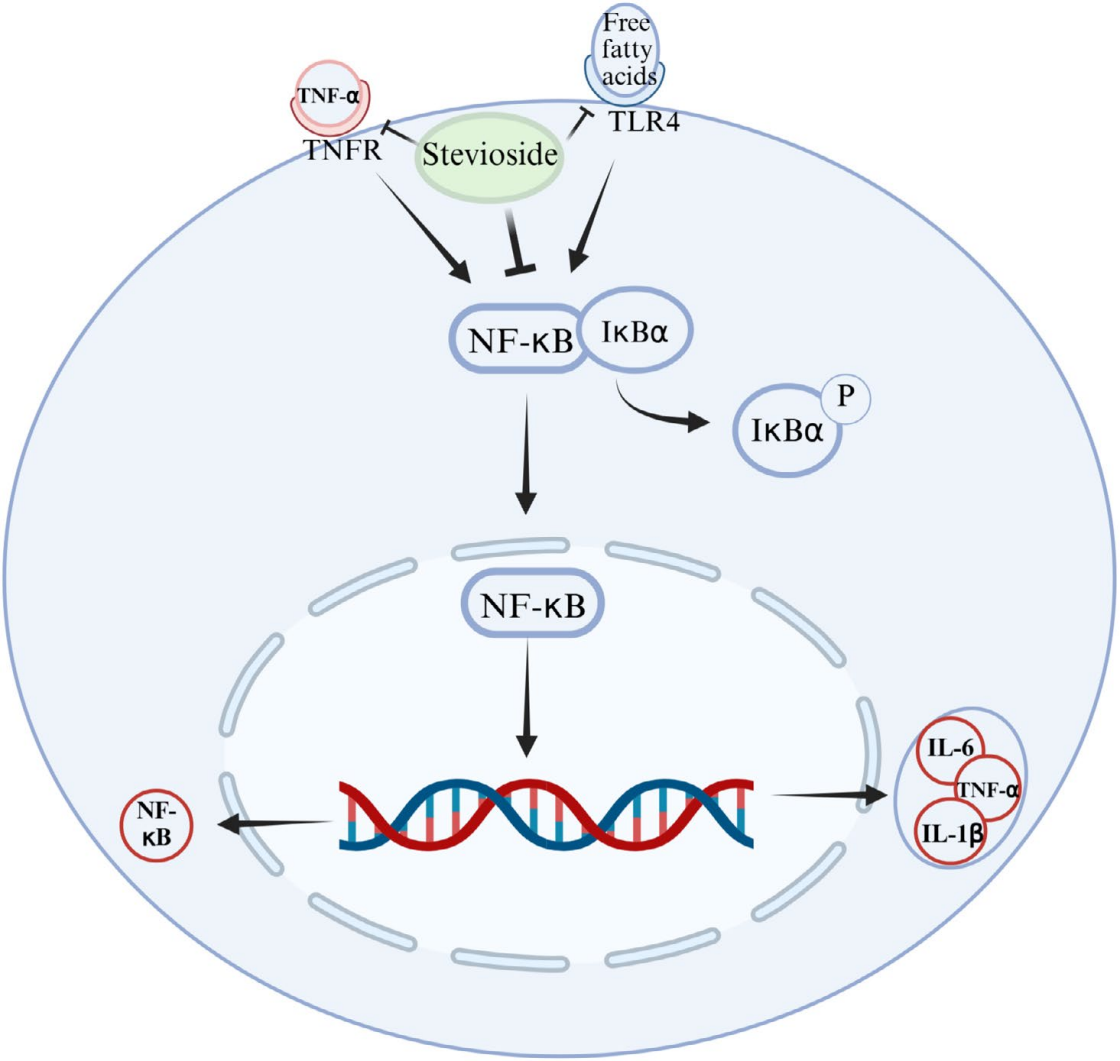
The potential anti‐inflammatory effect of SV on the liver. SV ameliorates metabolic dysfunction‐associated steatohepatitis potentially by interacting with upstream receptors TLR4 and TNFR1 (based on in silico/in vitro evidence) in the NF‐κB signaling pathway and suppressing NF‐κB nuclear translocation.

In summary, SV ameliorated MASLD by interacting with upstream receptors TLR4 and TNFR1 in the NF‐κB signaling pathway, and suppressing NF‐κB nuclear translocation (Figure 4). Although SV has demonstrated anti‐inflammatory potential, current evidence supporting SV's antagonistic activity against TLR4/TNFR1 was predominantly derived from experimental studies with heterogeneous models, interventions, and outcome definitions.

### 4.4 SV Exhibited Multifaceted Anti‐Fibrotic Properties

As MASLD progresses, hepatocytes are partially replaced by fibrotic scar tissue and undergo substantial alterations. Consequently, increasing attention has been directed toward anti‐fibrotic effects on ameliorating MASLD‐related fibrosis (Schwabe et al. 2020).

Within the hepatic microenvironment, activated hepatic stellate cells serve as the principal source of extracellular matrix production (Wells and Schwabe 2015). Transforming growth factor‐beta (TGF‐β) not only functions as the most potent profibrogenic cytokine but also acts as a critical driver of hepatic stellate cells activation and fibrogenesis (Latha et al. 2017). Some recent investigations have reported potential anti‐fibrotic effects of SV via modulation of the TGF‐β pathway. For instance, Casas‐Grajales, Alvarez‐Suarez, et al. (2019) demonstrated that SV administration (20 mg/kg BW) significantly reduced hepatic collagen deposition in thioacetamide‐induced rats, accompanied by downregulation of α‐smooth muscle actin expression and suppression of fibrotic progression. Further mechanistic investigations revealed that SV attenuated extracellular matrix production through direct downregulation of TGF‐β1 and inhibition of its downstream SMAD signaling pathway (suppressing p‐SMAD3, p‐JNK, and p‐p38 and upregulating inhibitory SMAD7) while concurrently inhibiting matrix metalloproteinase enzymatic activity. They also validated these findings using a coculture model of human hepatic stellate cells and VL‐17A cells to simulate hepatic fibrosis, in which SV (100 μM) exhibited similar anti‐fibrotic effects via inhibition of the TGF‐β pathway.

Stevia extracts demonstrated anti‐fibrotic effects on hepatic fibrosis. Administration of stevia extracts (100 mg/kg BW) effectively suppressed hepatic stellate cells activation and improved fibrotic manifestations through coordinated inhibition of TGF‐β, matrix metalloproteinases, and connective tissue growth factor, while maintaining expression of the anti‐fibrotic mediator SMAD7 (Ramos‐Tovar, Buendia‐Montaño, et al. 2019).

**FIGURE 5:**
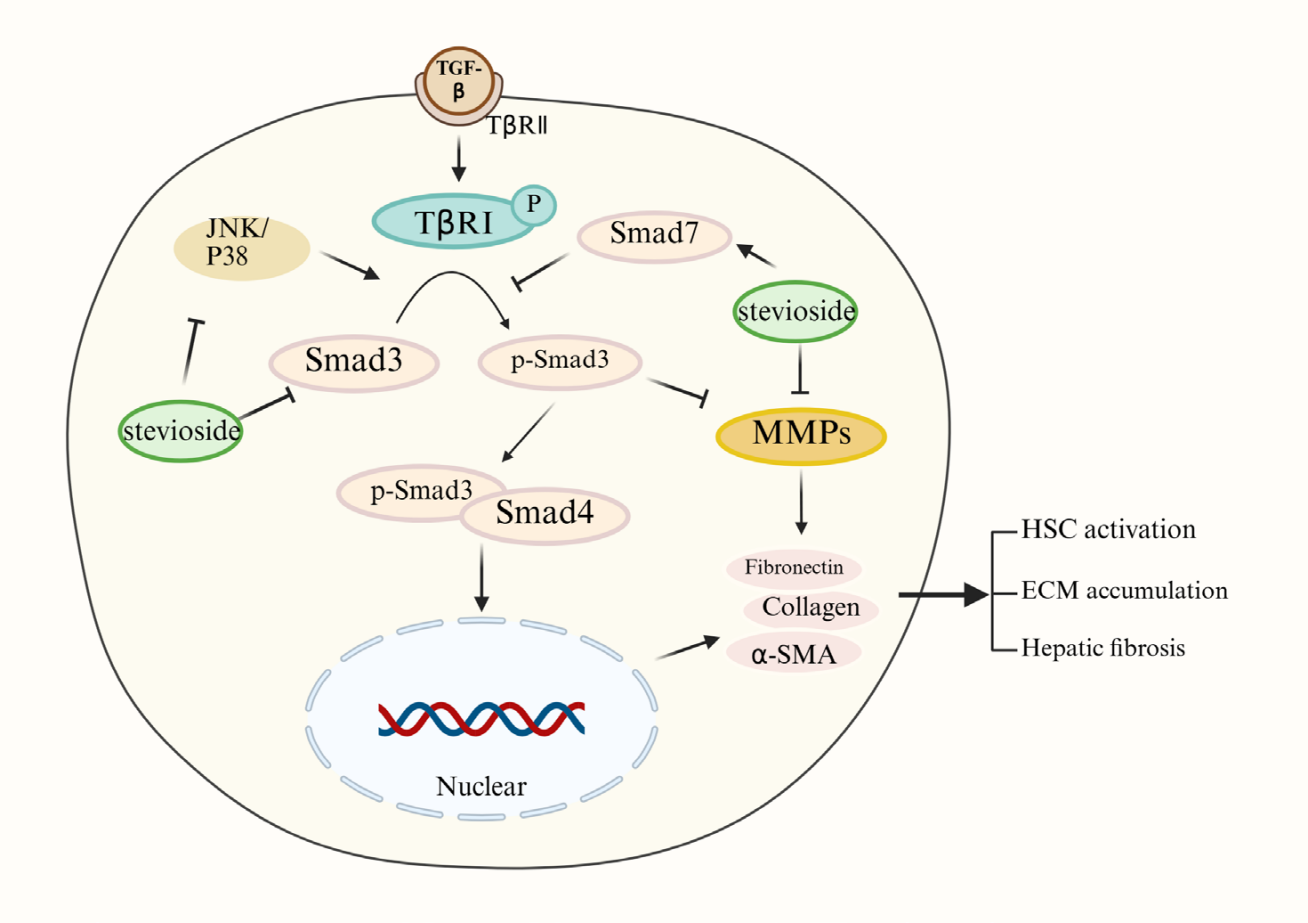
The potential anti‐fibrotic effects of SV on the liver. SV ameliorates hepatic fibrosis through dual mechanisms involving TGF‐β pathway inhibition and MMP suppression, thereby reducing hepatic stellate cells' activation/transition and subsequent extracellular matrix accumulation.

Therefore, SV could improve hepatic fibrosis through dual mechanisms involving inhibition of the TGF‐β pathway and suppression of matrix metalloproteinases, thereby reducing hepatic stellate cell activation/transition and extracellular matrix accumulation (Figure 5). However, the pathobiology of hepatic fibrosis involved complex interactions that were largely unclear, while evidence for SV remained limited, particularly regarding its interactions with the dynamic fibrotic microenvironment in MASLD.

### 4.5 Others

Other steviol glycosides, including rebaudioside A and rebaudioside D, were also observed to have similar effects of SV on MASLD (Curry and Roberts 2008; Myint et al. 2020; Nikiforov et al. 2013). For instance, rebaudioside A (10–100 mg/L) attenuated hepatic lipid accumulation by downregulating lipogenesis‐related genes and activating the AMPK pathway (Khan et al. 2021). Moreover, rebaudioside A (10–30 μM) mitigated CCl_4_‐induced oxidative stress in HepG2 cells by activating the NRF2 signaling pathway, enhanced antioxidant enzymes (HO‐1, NQO1), and reduced reactive oxygen species and malondialdehyde levels (Wang et al. 2018). Notably, rebaudioside A (194 mg/L) reduced hepatic steatosis, fibrosis, and endoplasmic reticulum stress in an HFD‐induced MASLD model (Xi et al. 2020), where this hepatoprotective effect was further validated in a thioacetamide‐induced liver injury model (Casas‐Grajales, Reyes‐Gordillo, et al. 2019). Likewise, rebaudioside D reduced hepatic lipid accumulation (triglycerides and cholesterol) by suppressing lipogenic pathways and attenuated oxidative stress via decreasing lipid peroxidation in obese mice (Morissette et al. 2024).

Recently, nano‐SV has been developed to enhance the bioavailability, stability, and functions of SV. One study showed that nano‐SV (20 mg/dL) significantly reduced hepatocyte apoptosis in streptozotocin‐induced diabetic rats and exhibited superior anti‐apoptotic effects when compared to SV (Mousavi‐Niri et al. 2023). Furthermore, the administration of nano‐SV (20 mg/dL) resulted in the amelioration of the disordered arrangement of hepatocytes in diabetic rats with MASLD, concomitant with improvements in blood glucose levels, anxiety, and memory impairment (Khakpai et al. 2023). The findings from all studies discussed above are summarized in Table 3.

**TABLE 3:** Effects of non‐SV steviol glycosides on MASLD.

Study ID	Experimental design details	Model	Main results	Potential mechanisms
Nikiforov et al. (2013)	Rebaudioside D (500–2000 mg/kg BW/day) in rats	Sprague–Dawley rats	No hepatic toxicity	Not reported
Curry and Roberts (2008)	Rebaudioside A (2500–10,000 mg/kg BW/day feeds) in rats	Wistar rats	No hepatic toxicity	Not reported
Myint et al. (2020)	Rebaudioside A (12 mg/kg BW/day) in mice	HFD‐streptozotocin induced mice	Reduced serum triglycerides, total cholesterol, and LDL‐C, increased HDL‐C; Enhanced antioxidant function of the liver.	Not reported
Khan et al. (2021)	Rebaudioside A (10–100 μg/mL) in mice	HFD and high‐carbohydrate diet‐induced obese mice (hepatic steatosis)	Reduced hepatic lipid accumulation; improved liver function markers.	Activation of AMPK pathway.
Wang et al. (2018)	Rebaudioside A (10–30 μM) in CCl_4_‐injured HepG2 cells	CCl_4_‐injured HepG2 cells	Protected against oxidative liver damage	NRF2 pathway activation. Promote HO‐1 and NQO1.
Xi et al. (2020)	Rebaudioside A (194 mg/L) in mice	HFD‐induced mice (hepatic steatosis)	Improved hepatic steatosis and fibrosis	Attenuated endoplasmic reticulum stress.
Casas‐Grajales, Reyes‐Gordillo, et al. (2019)	Rebaudioside A (20 mg/kg BW/day) in rats	Thioacetamide‐induced rats (hepatitis)	Inhibition of lipid peroxidation; downregulation of pro‐inflammatory factors; attenuation of liver fibrosis.	Activation of NRF2 pathway. Inhibition of NF‐κB pathway. Stabilization of collagen content.
Morissette et al. (2024)	Rebaudioside D (50 mg/kg BW/day) in mice	HFD/high‐sucrose diet‐induced obese mice (hepatic steatosis)	Reduced hepatic triglycerides and cholesterol; decreased lipid peroxidation.	Inhibit lipopolysaccharide‐binding protein.
Mousavi‐Niri et al. (2023)	Nano‐stevia (20 mg/dL) in rats	Streptozotocin‐induced diabetic rats	Reduced hepatocyte apoptosis and liver injury in diabetic rats.	Downregulation of PEPCK, upregulation of GCK genes.
Khakpai et al. (2023)	Nano‐SV (1 mL/kg BW/day) in streptozotocin‐induced diabetic rats	Streptozotocin‐induced diabetic rats (hepatic steatosis)	Restored normal liver tissue architecture in diabetic rats; improvements in blood glucose levels, anxiety, and memory impairment.	Not reported

Abbreviations: AMPK, AMP‐activated protein kinase; GCK, glucokina; HO‐1, heme oxygenase‐1; NF‐κB, nuclear factor kappa B; NQO1, NAD(P)H quinone dehydrogenase 1; Nrf2, nuclear factor erythroid 2‐related factor 2; PEPCK, phosphoenolpyruvate carboxykinase.

It is noteworthy that the MASLD animal model can simulate a specific stage in the development of clinical MASLD, but it cannot fully replicate the widespread metabolic dysfunction and all clinical symptoms observed in human patients. The MASLD models established by using streptozotocin and/or HFD have some limitations. While exposure to both streptozotocin and HFD increases liver fibrosis in mice, it could not result in more advanced fibrosis or hepatocellular carcinoma associated with MASLD (Lo et al. 2011). Biologically, while streptozotocin induces pancreatic β‐cell destruction and insulin deficiency in animal models, human MASLD is typically accompanied by hyperinsulinemia and insulin resistance, and the sustained activation of insulin/IGF‐1 signaling is considered critical for hepatocarcinogenesis (Heydemann 2016). Besides, the MASLD animal model constructed through a specialized diet can accelerate metabolic dysfunction‐associated steatohepatitis and hepatocellular carcinoma; however, such a model may not replicate characteristics of metabolic dysfunction in humans including insulin resistance, systemic inflammation, and obesity (Farrell et al. 2019). Whereas diets high in fat or carbohydrates could induce hepatic steatosis without progressing to steatohepatitis, the methionine‐ and choline‐deficient (MCD) diets can effectively build liver fibrosis models. However, the MCD model is associated with significant weight loss and reduced serum insulin levels in mice (Ibrahim et al. 2016). Therefore, caution is needed when extrapolating these animal results to humans. Nevertheless, animal models remain crucial for exploring the pathophysiology of MASLD and potential therapeutic strategies to ultimately help prevent and treat MASLD in human patients (Jeong et al. 2024).

## 5 Discussion

SV, as a natural sweetener widely used in the food industry, has garnered increasing attention for its hepatoprotective potential, including lipid metabolism modulation, antioxidant, anti‐inflammatory, and anti‐fibrotic effects. These properties of SV may make it a promising approach to the potential prevention and treatment of MASLD.

In clinical trials, stevia supplementation was observed to enhance oxygen radical absorbance capacity in overweight individuals with baseline antioxidant deficiencies and to elevate circulating anti‐inflammatory cytokine IL‐10 levels, indicating its anti‐inflammatory potential. However, the effects of SV on lipid profiles and hepatic function markers were inconsistent with the majority of preclinical findings, highlighting an important translational gap. These discrepancies may be attributed to inherent limitations in clinical trial designs, including small sample sizes, short intervention durations, and the lack of direct hepatic tissue sampling for mechanistic validation. Moreover, the doses used in preclinical models significantly differed from those in clinical dosing regimens. SV was administered as a beverage additive at doses substantially lower than the recommended daily intake thresholds set by global food safety authorities (i.e., 4 mg/kg BW/day by European Food Safety Authority (Younes et al. 2023) vs. < 1 mg/kg BW/day in these two trials), mainly due to the aim of controlling its intense sweetness in beverages. This contrasts with preclinical studies, in which SV was administered as a therapeutic supplement at much higher doses (up to 2500 mg/kg BW/day) to explore hepatoprotective mechanisms. Subtherapeutic dosing, along with design limitations, may thus bias the effects of SV in clinical trials on MASLD (Kanuri and Bergheim 2013).

Therefore, this dose discrepancy raises a fundamental question in translational research: how to compare animal doses with realistic human intake? The majority of preclinical evidence for SV came from rodent studies using doses of 20–100 mg/kg/day and, in some mechanistic studies, up to 500 mg/kg BW/day (Kurek et al. 2020). Applying standard body surface area normalization (rat‐to‐human conversion factor ~6.2), a rat dose of 200 mg/kg equates to a human equivalent dose of approximately 32 mg/kg. For a 60 kg individual, this translates to a daily intake of ~1920 mg—far exceeding the JECFA/EFSA acceptable daily intake of 0–12 mg/kg/day (Nikiforov et al. 2013). This underscores the fact that the supraphysiological doses in animal models are tools for mechanistic discovery, not recommendations for human consumption. Thus, results from mechanistic insights could provide biological plausibility, requiring more translational studies for further exploration and validation.

Some limitations for preclinical studies should be acknowledged. First, most evidence came from rodent models, which failed to fully replicate the genetic, metabolic, and immune heterogeneity of human MASLD pathophysiology (Fang et al. 2022). For instance, the methionine‐choline deficient model fails to recapitulate MASLD‐associated metabolic derangements (Wang et al. 2019). Animal models using HFD regimens also exhibited significant between‐group variability, complicating comparative analyses (Baumgardner et al. 2008). Second, the mechanisms of SV required more in‐depth elucidation. Hepatocyte injury in MASLD manifests characteristic pathological features, including endoplasmic reticulum stress, dysfunctional unfolded protein response, and inflammasome activation (Puri et al. 2008; Szabo and Petrasek 2015), while the inflammasome serves as an interface between initial metabolic stress and subsequent hepatic fibrogenesis (Csak et al. 2011). Nevertheless, the mechanistic basis underlying SV's modulation of these interconnected pathways remained largely unknown. In addition, the pharmacokinetic properties of SV and its potential food–drug interactions were insufficiently characterized (Thøgersen et al. 2018). Although in vivo studies used doses of 20–100 mg/kg BW/day, the low oral bioavailability of SV and its rapid conversion to steviol via gut metabolism created significant uncertainty regarding its effective doses in the liver. Furthermore, although emerging evidence indicated significant alterations in gut microbiota in MASLD patients (Loomba et al. 2017), no evidence for the interplay between SV, microbial dysbiosis, and MASLD pathogenesis was available.

Other limitations for our review are also noted. First, the literature search was restricted to English‐language articles indexed in PubMed and Web of Science, potentially excluding relevant studies published in other databases or in non‐English languages. Some preclinical studies utilized animal models of secondary hepatic steatosis induced by metabolic disorders (e.g., diabetes) that could not reflect the pure MASLD pathogenesis. To capture the relevant evidence as much as possible, we included these complementary studies aiming to best elucidate the potential effects of SV on MASLD. Similarly, we included studies using stevia extracts and other types of steviol glycosides for intervention to retrieve the maximum available evidence. Histological evaluation is operationally expedient for assessing MASLD progression in preclinical research settings. Some preclinical investigations used hematoxylin–eosin staining to validate hepatic pathology, yet they uniformly omitted evaluation of MASLD activity scores. This was an important methodological limitation in current preclinical MASLD studies. Furthermore, the interpretation of mechanistic discrepancies across studies was constrained by the methodological heterogeneity and risk of incomplete data reporting in the original publications. Therefore, our results should be interpreted with caution as a preliminary framework to guide future investigations.

As a sugar substitute, SV is a promising candidate for nutritional strategies targeting MASLD. Current evidence indicates that SV may play a beneficial role by improving lipid metabolism, enhancing anti‐inflammatory and antioxidant capacity, and attenuating hepatic fibrosis. It should be noted, however, that many of these positive outcomes, particularly in preclinical studies, were observed with megadoses rather than typical dietary levels of intake. However, the clinical evidence supporting these effects remains sparse and limited. Therefore, further clinical studies are strongly indicated to validate whether SV can be an effective strategy for addressing MASLD.

The present findings indicate that diverse steviol glycosides (e.g., rebaudioside A, D, stevioside) undergo gut microbial hydrolysis to steviol without generating toxic metabolites, supporting flexible use in food formulations for optimizing taste and functionality while aligning with established safety approvals. Preclinical evidence suggested multi‐target bioactivities, positioning these glycosides as potential bioactive ingredients beyond sugar replacement. From a public health perspective, they could serve as safe substitutes to reduce sugar intake and thus prevent obesity and MASLD. However, it is important to note the vast difference between animal testing doses and human intake levels. Dietary guidance should thus emphasize the overall intake of SV from diets and supplements, rather than standalone therapeutic use.

## 6 Conclusion

Preclinical studies suggest that stevioside could modulate hepatic lipid metabolism, oxidative stress, inflammation, and fibrotic signaling; however, clinical evidence remains limited, and MASLD‐specific endpoints have not yet been rigorously tested in humans. To conclude, SV can serve as a promising dietary strategy for potential prevention and treatment of MASLD if its hepatoprotective effects could be further confirmed and validated in clinical trials and population studies.

## Author Contributions

**Kangjun Li:** writing – original draft, investigation, validation, conceptualization, methodology. **Changfa Zhang:** conceptualization, methodology, writing – review and editing. **Lili Kang:** writing – review and editing, investigation. **Yunping Mu:** writing – review and editing. **Zemin Li:** writing – review and editing, methodology. **Jingyi Zhang:** writing – review and editing. **Guowei Li:** conceptualization, methodology, funding acquisition, resources, writing – review and editing, project administration.

## Funding

This study was funded by the National Natural Science Foundation of China (82473612), the Natural Science Foundation of Guangdong Province of China (2025A1515010779), the Science Foundation of Guangdong Second Provincial General Hospital (YY2018‐002), and the Young Top Talent Project in Special Support Plan for Training High‐level Talents in Guangdong (0720240244).

## Ethics Statement

The authors have nothing to report.

## Conflicts of Interest

The authors declare no conflicts of interest.

## Supporting information


**Data S1:** PRISMA 2020 checklist.

**Figure S1:** Summary of risk of bias for the included clinical trials using RoB 2.0.
**Table S1:** Detailed search strategies used for the database search.
**Table S2:** List of the 40 studies included for our review.

## Data Availability

The data that supports the findings of this study are available in the Supporting Information of this article.
