# Supplementary material for: Effects and Mechanisms of Stevioside on Metabolic Dysfunction‐Associated Steatotic Liver Disease
Source: Food Sci Nutr. 2026 May 27;14(6):e71945. doi: 10.1002/fsn3.71945 (PMC13239865; doi:10.1002/fsn3.71945)
Supplement: Supplementary file 2 — Figure S1: Summary of risk of bias for the included clinical trials using RoB 2.0. Table S1: Detailed search strategies used for the database search. Table S2: List of the 40 studies included for our review. [file FSN3-14-e71945-s001.docx]

**Table S1.** Detailed search strategies used for the database search

| **PubMed** | | |
| --- | --- | --- |
| **Step** | **Detailed Search strategies** | **No. of records** |
| #1 | "stevia"[Mesh] | 695 |
| #2 | stevia*[Title/Abstract] | 1,275 |
| #3 | "steviol" [Supplementary Concept] | 253 |
| #4 | "stevioside" [Supplementary Concept] | 501 |
| #5 | "rebaudioside A" [Supplementary Concept] | 181 |
| #6 | "rebaudioside D" [Supplementary Concept] | 18 |
| #7 | "rebaudioside M" [Supplementary Concept] | 8 |
| #8 | "steviol glycoside*"[Title/Abstract] | 542 |
| #9 | "Non-alcoholic fatty liver disease" [Mesh] | 28,344 |
| #10 | "Non-alcoholic fatty liver disease" [Title/Abstract] | 19,462 |
| #11 | "Metabolic dysfunction-associated steatotic liver disease" [Title/Abstract] | 2,583 |
| #12 | "Lipid metabolism" [Mesh] | 96,409 |
| #13 | "Liver fibrosis" [Title/Abstract] | 25,972 |
| #14 | "Lipid Metabolism" [Title/Abstract] | 68,997 |
| #15 | "Lipid Metabolic" [Title/Abstract] | 3,929 |
| #16 | "Oxidative stress" [Mesh] | 185,352 |
| #17 | "Oxidative stress" [Title/Abstract] | 298,825 |
| #18 | "Hepatic inflammation" [Title/Abstract] | 3,577 |
| #19 | "liver"[Mesh] | 487,098 |
| #20 | "liver"[Title/Abstract] | 1,016,776 |
| #21 | "hepatic"[Title/Abstract] | 358,955 |
| #22 | #1 OR #2 OR #3 OR #4 OR #5 OR #6 OR #7 OR #8 | 1,692 |
| #23 | #9 OR #10 OR #11 OR #12 OR #13 OR #14 OR #15 | 194,722 |
| #24 | #16 OR #17 OR #18 #19 OR #20 OR #21 | 1,468,090 |
| #25 | #23 OR #24 | 1,670,779 |
| #26 | #22 AND #25 | 160 |
| **Web of Science** | | |
| #1 | TS=(Stevia* OR steviol* OR stevioside OR rebaudioside A OR rebaudioside D OR rebaudioside M OR steviol glycoside*) | 21,288 |
| #2 | TS=((Non-alcoholic fatty liver disease OR Metabolic dysfunction-associated steatotic liver disease) OR (Lipid metabolism OR Liver fibrosis OR Oxidative stress OR Hepatic inflammation) OR (liver OR hepatic)) | 3,638,154 |
| #3 | #1 AND #2 | 772 |

**Figure S1.** Summary of risk of bias for the included clinical trials using RoB 2.0.

**
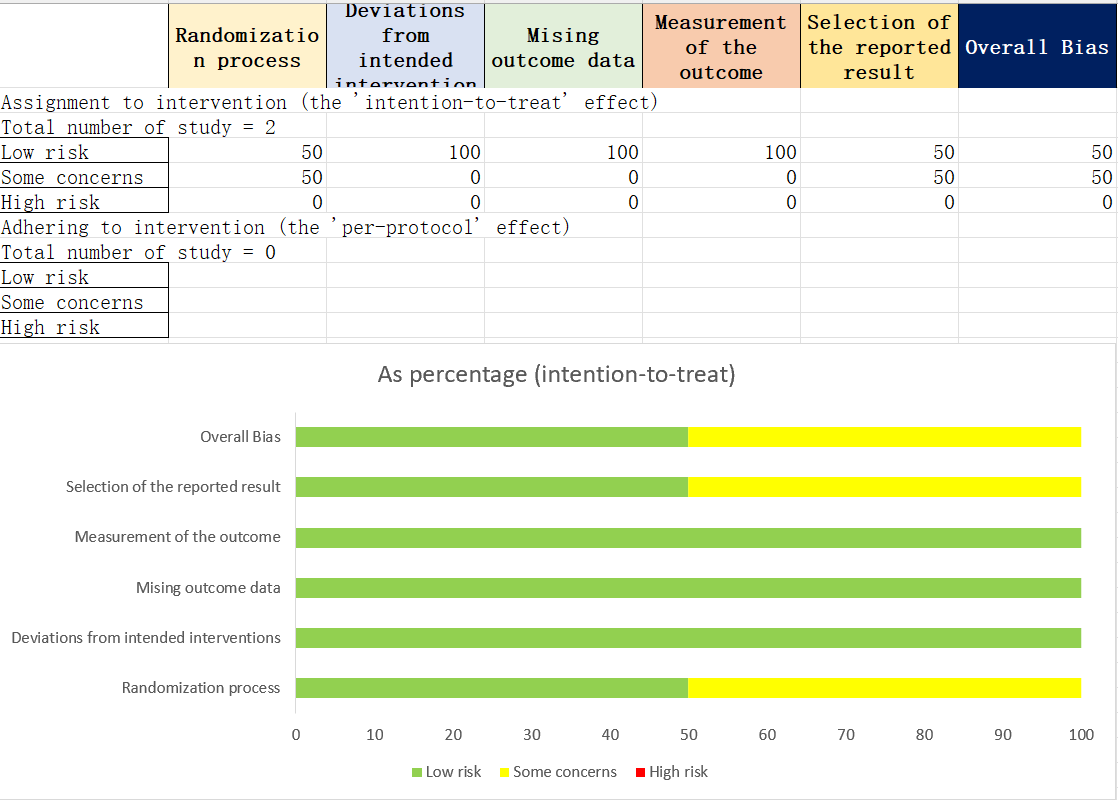
Table S2.** List of the 40 studies included for our review

| **Study ID^1^** | **Reference** |
| --- | --- |
| Ramos-Tovar, 2018 | Stevia rebaudiana tea prevents experimental cirrhosis via regulation of NF-κB, Nrf2, transforming growth factor beta, Smad7, and hepatic stellate cell activation. *Phytother Res,* 32(12): p. 2568-2576. |
| Park, 2022 | Stevia and Stevioside Attenuate Liver Steatosis through PPARα-Mediated Lipophagy in db/db Mice Hepatocytes. *Antioxidants (Basel)*, 11(12). |
| Mousavi-Niri, 2023 | Nano-Stevia reduces the liver injury caused by streptozotocin (STZ)-induced diabetes in rats by targeting PEPCK/GCK genes, INSR pathway and apoptosis. *J Diabetes Metab Disord，* 22(2):1519-1529. |
| Ramos-Tovar, 2018 | Stevia Prevents Acute and Chronic Liver Injury Induced by Carbon Tetrachloride by Blocking Oxidative Stress through Nrf2 Upregulation. *Oxid Med Cell Longev*, p. 3823426. |
| Khakpai, 2023 | Intra-gastrically administration of Stevia and particularly Nano-Stevia reversed the hyperglycemia, anxiety, and memory impairment in streptozotocin-induced diabetic rats. *Physiol Behav*， 263:114100. |
| Chaudhary, 2017 | Hydroalcoholic extract of Stevia rebaudiana bert. leaves and stevioside ameliorates lipopolysaccharide induced acute liver injury in rats. *Biomed Pharmacother*, 95: p. 1040-1050 |
| Holvoet, 2015 | Stevia-derived compounds attenuate the toxic effects of ectopic lipid accumulation in the liver of obese mice: a transcriptomic and metabolomic study. *Food Chem Toxicol*, 77: p. 22-33. |
| Saadi, 2024 | Therapeutic potential of aquatic Stevia extract in alleviating endoplasmic reticulum stress and liver damage in streptozotocin-induced diabetic rats. *Mol Biol Rep*, 51(1): p. 993. |
| Nikiforov, 2013 | Metabolism and toxicity studies supporting the safety of rebaudioside D. Int J *Toxicol*, 32(4):261-73. |
| Potočnjak, 2017 | Stevia and stevioside protect against cisplatin nephrotoxicity through inhibition of ERK1/2, STAT3, and NF-κB activation. *Food Chem Toxicol*, 107(Pt A): p. 215-225. |
| Casas-Grajales, 2019 | Antioxidant and immunomodulatory activity induced by stevioside in liver damage: In vivo, in vitro and in silico assays. *Life Sci*, 224: p. 187-196. |
| Morissette, 2024 | Rebaudioside D decreases adiposity and hepatic lipid accumulation in a mouse model of obesity. *Sci Rep*, 14(1):3077. |
| Ranjbar, 2022 | A comparison of the effects of Stevia extract and metformin on metabolic syndrome indices in rats fed with a high-fat, high-sucrose diet. *J* *Food Biochem*, 44(8): p. e13242. |
| Shivanna, 2013 | Antioxidant, anti-diabetic and renal protective properties of Stevia rebaudiana. *J Diabetes Complications*, 27(2): p. 103-13. |
| Ramos-Tovar, 2019 | An aqueous extract of Stevia rebaudiana variety Morita II prevents liver damage in a rat model of cirrhosis that mimics the human disease. *Ann Hepatol*, 18(3): p. 472-479. |
| Kurek, 2023 | Steviol glycosides from Stevia rebaudiana Bertoni mitigate lipid metabolism abnormalities in diabetes by modulating selected gene expression - An in vivo study. *Biomed Pharmacother*, 166: p. 115424. |
| Aghajanyan, 2017 | Antihyperglycemic and Antihyperlipidemic Activity of Hydroponic Stevia rebaudiana Aqueous Extract in Hyperglycemia Induced by Immobilization Stress in Rabbits. *Biomed Res Int*, 2017:9251358. |
| Khan, 2021 | Pharmacological Approaches to Attenuate Inflammation and Obesity with Natural Products Formulations by Regulating the Associated Promoting Molecular Signaling Pathways. *Biomed Res Int*, 2021:2521273. |
| Curry, 2008 | Subchronic toxicity of rebaudioside A. *Food Chem Toxicol*, 46 Suppl 7:S11-20. |
| Kurek, 2020 | Steviol Glycosides Supplementation Affects Lipid Metabolism in High-Fat Fed STZ-Induced Diabetic Rats. *Nutrients,* 13(1):112. |
| Ghanta, 2007 | Oxidative DNA damage preventive activity and antioxidant potential of Stevia rebaudiana (Bertoni) Bertoni, a natural sweetener. *J Agric Food Chem*, 55(26):10962-7. |
| Wang, 2018 | New application of the commercial sweetener rebaudioside a as a hepatoprotective candidate: Induction of the Nrf2 signaling pathway. *Eur J Pharmacol*, 822:128-137. |
| Bracht, 1985 | Effects of Stevia rebaudiana natural products on rat liver mitochondria. *Biochem Pharmacol*, 34(6):873-82. |
| Ferreira, 2006 | Comparative effects of Stevia rebaudiana leaves and stevioside on glycaemia and hepatic gluconeogenesis. *Planta Med*, 72(8):691-6. |
| Park, 2010 | Stevia rebaudiana Bertoni extract supplementation improves lipid and carnitine profiles in C57BL/6J mice fed a high-fat diet. *J Sci Food Agric*, 90(7):1099-105. |
| Casas-Grajales, 2019 | Rebaudioside A administration prevents experimental liver fibrosis: an in vivo and in vitro study of the mechanisms of action involved. *J Appl Toxicol*, 39(8):1118-1131. |
| Ramos-Tovar,  2019 | Stevia prevents experimental cirrhosis by reducing hepatic myofibroblasts and modulating molecular profibrotic pathways. *Hepatol Res*, 49(2):212-223. |
| El-Hadary, 2021 | Safely effective hypoglycemic action of stevia and turmeric extracts on diabetic Albino rats. *J Food Biochem*, 45(1):e13549. |
| Casas-Grajales, 2019 | Stevioside inhibits experimental fibrosis by down-regulating profibrotic Smad pathways and blocking hepatic stellate cell activation. *Basic Clin Pharmacol Toxicol*, 124(6):670-680. |
| Jia, 2019 | Attenuation of high-fat diet-induced fatty liver through PPARα activation by stevioside. *Journal of Functional Foods*, 57: p. 392-398. |
| Liu,  2022 | Dietary Stevia Residue Extract Supplementation Improves Antioxidant Capacity and Intestinal Microbial Composition of Weaned Piglets. *Antioxidants (Basel)*, 11(10):2016. |
| Tang, 2023 | Improving gut functions and egg nutrition with stevia residue in laying hens. *Poult Sci*, 103(2):103324. |
| Pirgozliev, 2021 | Feeding dry stevia leaf (Stevia rebaudiana) or xylanase improves the hepatic antioxidative status of broiler chickens. *Res Vet Sci*, 136:227-229. |
| Rotimi, 2018 | Stevioside modulates oxidative damage in the liver and kidney of high fat/low streptozocin diabetic rats. *Heliyon*, 4(5):e00640. |
| Zafrilla, 2021 | Biological effects of stevia, sucralose and sucrose in citrus-maqui juices on overweight subjects. *Food Funct*, 12(18):8535-8543. |
| Thøgersen, 2018 | In vitro effects of rebaudioside A, stevioside and steviol on porcine cytochrome p450 expression and activity. *Food Chem*, 258:245-253. |
| Xi, 2020 | Rebaudioside affords hepatoprotection ameliorating sugar sweetened beverage- induced nonalcoholic steatohepatitis. *Sci Rep*, 10(1):6689. |
| Vaško, 2014 | Comparison of some antioxidant properties of plant extracts from Origanum vulgare, Salvia officinalis, Eleutherococcus senticosus and Stevia rebaudiana. *In Vitro Cell Dev Biol Anim*, 50(7):614-22. |
| Xiong, 2025 | Dietary steviol glycosides mixture supplementation modulates the gene expression of gut chemoreceptors and enhances the antioxidant capacity in weaned piglets. *Porcine Health Manag*, 11(1):6. |
| Almiron-Roig, 2023 | Impact of acute consumption of beverages containing plant-based or alternative sweetener blends on postprandial appetite, food intake, metabolism, and gastro-intestinal symptoms: Results of the SWEET beverages trial. *Appetite*, 184:106515. |

^1^ Shown as first author, publication year.
